# PRL-3 overexpression in epithelial cells is induced by surrounding stromal fibroblasts

**DOI:** 10.1186/1476-4598-8-46

**Published:** 2009-07-08

**Authors:** David G Molleví, Alvaro Aytes, Mireia Berdiel, Laura Padullés, Maria Martínez-Iniesta, Xavier Sanjuan, Ramon Salazar, Alberto Villanueva

**Affiliations:** 1 Translational Research Laboratory, Institut Català d'Oncologia, Institut d'Investigació Biomèdica de Bellvitge (IDIBELL), 08907 L'Hospitalet de Llobregat, Barcelona, Spain; 2 Herbert Irving Comprehensive Cancer Center, Columbia University, USA; 3 Department of Pathology, Hospital Universitari de Bellvitge, Institut d'Investigació Biomèdica de Bellvitge (IDIBELL), 08907 L'Hospitalet de Llobregat, Barcelona, Spain; 4 Department of Medical Oncology, Institut Català d'Oncologia, Institut d'Investigació Biomèdica de Bellvitge (IDIBELL), 08907 L'Hospitalet de Llobregat, Barcelona, Spain

## Abstract

We isolate and culture carcinoma-associated fibroblasts (CAFs) from primary tumour (CAFpt), CAFs from corresponding synchronous liver metastasis (CAFlm) as well as normal colonic fibroblasts (NCF) from the same patient. From these cultures, conditioned media (CM) was obtained. Culture of a wide panel of colorectal and pancreatic cell lines in CM from CAFlm resulted in overexpression of mRNA PRL-3 and higher overexpression in CAFs than in non-activated fibroblasts. Moreover PRL-3 mRNA expression correlates with expression of α-SMA and deposition of collagen fibrils in the stroma. We demonstrate that products secreted by CAFs trigger PRL-3 overexpression in cancer cells. Identification of these factors may contribute to new stroma-targeted therapies for desmoplastic tumours.

## Introduction

In many epithelial tumours inclouding colorectal, the relevance of PRL-3 phosphatase to the tumorigenic process of distant dissemination is well documented [1-5]. While some manuscripts adress the downstream pathways in which PRL-3 could influence the processes of migration and invasion [6,7], there is a complete lack of knowledge of the events upstream PRL-3.

In colorectal tumorigenesis, PRL-3 was identified as a molecule consistently overexpressed in hepatic metastases. These tumours are characterised by a strong desmoplastic reaction, consisting predominantly of carcinoma-associated fibroblasts (CAFs) and collagen I fibrils, which are also a hallmark of colorectal adenocarcinomas (CRC). In the case of primary tumours, the desmoplastic reaction contributes to the spreading of tumoral epithelial cells. But for liver metastasis, the biological significance of desmoplasia is still unclear. Some authors consider desmoplasia as a wall to keep tumour cells under control. If desmoplasia is a mechanism of walling off or is a reaction to promote spreading of epithelial cells will depend probably on the composition of extracellular matrix (ECM) and cells responsible for the deposition of ECM components. Therefore, cross-talk between epithelial and mesenchymal cells could influence the behaviour of the tumour population.

In this study we report modulation of PRL-3 expression in CRC cell lines dependant on soluble factors released into fibroblast conditioned media (CM). Moreover, we describe an association between PRL-3 overexpression in epithelial cells and desmoplastic reaction in human samples.

## Findings

### PRL-3 is overexpressed in different colorectal and pancreatic cell lines when cultured in conditioned media derived from carcinoma-associated fibroblasts from liver metastasis

**Figure 1:**
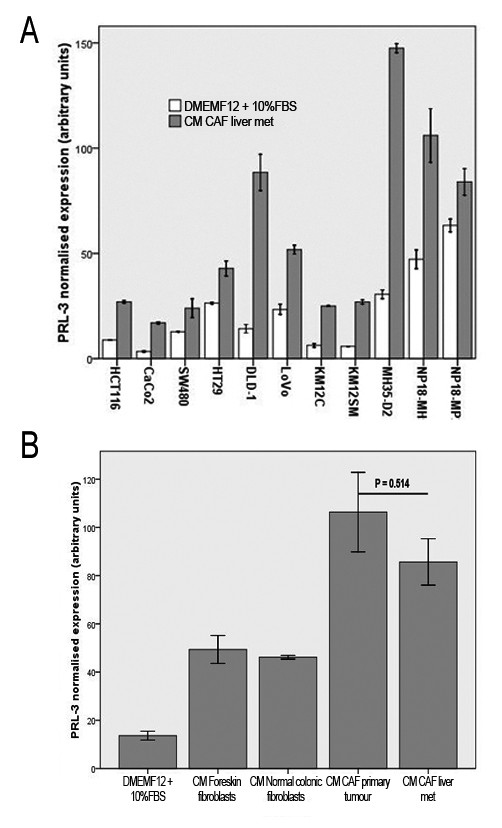
**A: normalised *PRL-3 *mRNA expression of colorectal (HCT-116, CaCo2, HT-29, DLD-1, LoVo, KM12C, KM12SM, SW480 and MH35-D2) and pancreatic cell lines (NP18-MP2 and NP18-MH5) cultured in DMEMF12 + 10% FBS or conditioned medium (CM) obtained from carcinoma-associated fibroblasts derived from a liver metastasis (CAFlm)**. Total RNA were retrieved from cellular pellets using TrizOL Reagent method. cDNA was obtained using recombinant ribonuclease inhibitor RNAse OUT and M-MLV retrotranscriptase. Quantitative real-time RT-PCR analyses were performed using the Light-Cycler 2.0 Roche System and LightCycler FastStart DNA Master SyBR Green I kit (Roche). For normalisation of *PRL-3 *expression levels we analysed expression of GAPDH. Oligonucleotides sequences are provided upon request. All of the experiments were performed in triplicate using two different retrotranscriptions. B: normalised *PRL-3 *mRNA expression of DLD-1 cells cultured in conditioned media (CM) derived from fibroblasts and carcinoma-associated fibroblasts. We obtained matched fibroblasts cultures from fresh surgical specimens from one patient with colorectal carcinoma with synchronous hepatic metastasis under the supervision of the Ethics Committee of the Hospital Universitari de Bellvitge. We isolated normal colonic fibroblasts (NCF) from the normal colonic mucosa at least 5 cm from the surgical margin, carcinoma-associated fibroblasts (CAFpt) from the primary tumour and carcinoma-associated fibroblasts (CAFlm) from the synchronous metastasis. We also obtained foreskin fibroblasts from another patient. All tissues were minced into small fragments and washed with Hanks Balanced Salt Solution (Gibco) and filtered with a 70 μm cell strainer (BD). Then fibroblasts and CAF's were cultured in Dulbecco's modified Eagle's F12 medium (Lonza), containing 10% fetal bovine serum (Gibco) and penicillin/streptomycin. After five passages, cells in confluence were harvested and cultures were used to make CM. Thus, fibroblasts or CAF's were grown to confluence in DMEMF12 containing 10% FBS. Then were washed twice in PBS and incubated for 48 h in DMEMF12 without FBS. Next, fibroblasts/CAFs cultures were washed twice in PBS and incubated for 48 h in DMEMF12 containing 10% FBS. CM was centrifuged 5 minutes at 3000 rpm and filtered in 22 μm units and stored at -80°C until use. Therefore, CM from activated myofibroblasts (CAFpt, CAFlm) produces a higher stimulation of mRNA PRL-3 than CM from normal colonic fibroblasts from the same patient. As a control we used DMEMF12 + 10% FBS. In all panels, error bars indicate standard deviation corresponding to three different experiments.

We cultured a wide panel of colorectal and pancreatic cell lines, derived from primary tumours and secondary sites for 24 hours in their standard culture medium (DMEMF12 + 10%FBS) or in conditioned medium obtained from CAFs derived from a CRC liver metastasis. CM was produced as follows: CAFs were grown to confluence and CM was harvested after 48 hours of culture, centrifuged to remove cellular debris and filtered. PRL-3 mRNA expression was examined by real-time Q-PCR in nine colorectal and two pancreatic cell lines. All colorectal cell lines exhibited low levels of mRNA PRL-3 expression when cultured in standard DMEMF12 medium plus 10%FBS. On the other hand, pancreatic cell lines displayed moderate levels. But interestingly, as shown in figure 1A, both cell lineages increased PRL-3 mRNA expression when cultured in CM derived from CAFs, with the highest fold change observed in DLD-1 cells (6.479 fold). We hypothesised that products released by the stroma interact with receptors in the epithelial compartment to induce PRL-3 overexpression. Since our colorectal cell panel include almost all genetic backgrounds, this cross-talk may be a common event in CRC.

### CM from various fibroblasts lineages produces differential mRNA PRL-3 expression

**Figure 2:**
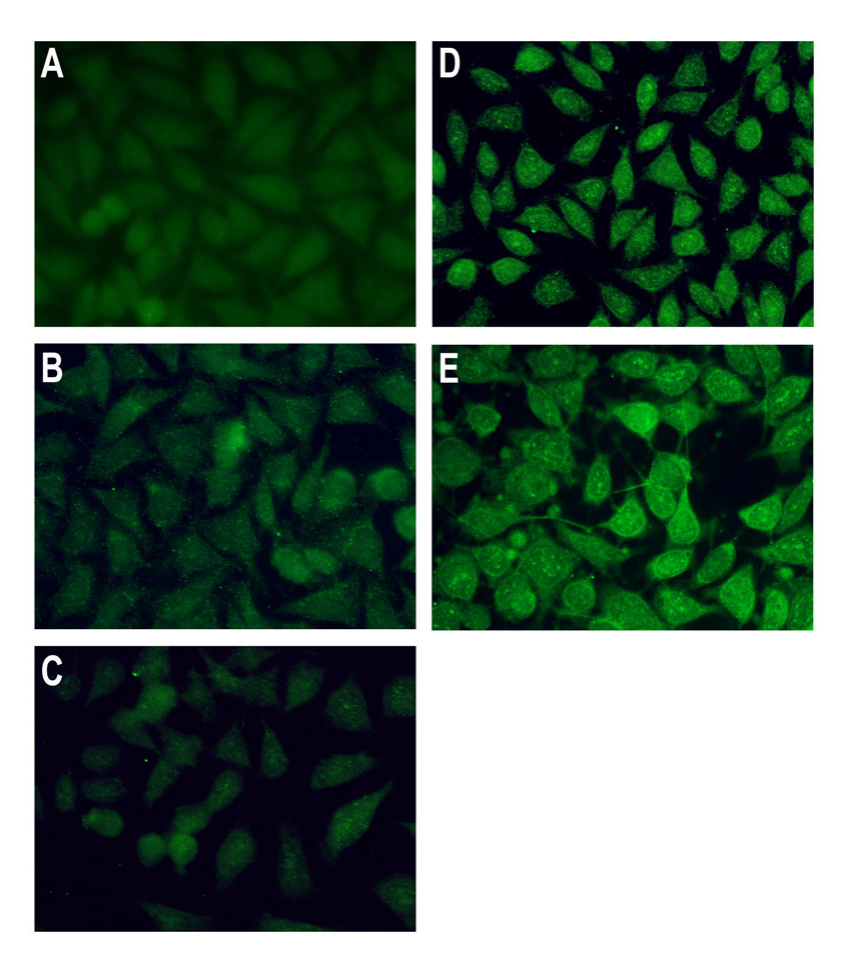
**Immunostaining of PRL-3 (Abcam, Cambridge, UK) in DLD-1 cells cultured in DMEMF12 + 10% FBS (A), CM from foreskin fibroblasts (B), CM from normal colonic fibroblasts (C), CM from CAFs from a primary tumour (D), CM from CAFs from liver metastasis (E)**. Briefly, cells were fixed 5 min. in ice cold methanol, washed in PBS and incubated for 1 hour in blocking buffer 1:5 in PBS (horse serum and goat serum, 1:1 v/v). Then, overnight incubation with rabbit polyclonal antibody against PRL-3 (Sigma Aldrich). After washing steps, 1 hour incubation with Goat anti rabbit IgG (H+L) FITC conjugated (Jackson).

We cultured DLD-1 cells for 24 h in CM, produced as described above, obtained from Foreskin fibroblasts (FF) and Normal colonic fibroblasts (NCF), metastasic primary tumour CAFs (CAFpt) and liver metastasis CAFs (CAFlm) from the same patient (matched samples). As shown in figure 1B, all CM induced significant (P < 0.05) overexpression of mRNA PRL-3 measured by Q-PCR compared with DMEMF12 control. Analysis of statistical significance used the ANOVA test and Tukey *post hoc*. Moreover, it is possible to put expression values in two groups according to activated (CAFpt and CAFlm; approximately 7 fold change in relation to control DMEMF12 + 10% FBS) or non-activated fibroblasts (FF and NCF; approximately 3.5 fold change). Comparing non activated fibroblasts, normal colonic fibroblasts (from a patient with colorectal cancer) and foreskin fibroblasts exerts the same effect on mRNA PRL-3 expression (P = 0.983). Same trend is observed between CAFpt and CAFlm (P = 0.514). Interestingly, comparing results from matched samples, conditioned media from NCF induced significantly (P = 0.036) lower levels of mRNA PRL-3 than CM from activated fibroblasts of the same patient. Accordingly, we conclude that CM obtained from CAFs more efficiently induce the overexpression of phosphatase PRL-3 than non-activated fibroblasts. The high expression values obtained with CAFpt are in agreement with a previous report [8] demonstrating high mRNA PRL-3 expression in primary Stage III tumours that developed distant metastasis. As shown in figure 2, this results were confirmed at the protein level in DLD-1 cells stimulated with CM from CAFpt and CAflm, reaching the highest intensity in cells cultured with CM CAFlm (figure 2E).

### mRNA PRL-3 expression levels correlates with the degree of desmoplasia

**Figure 3:**
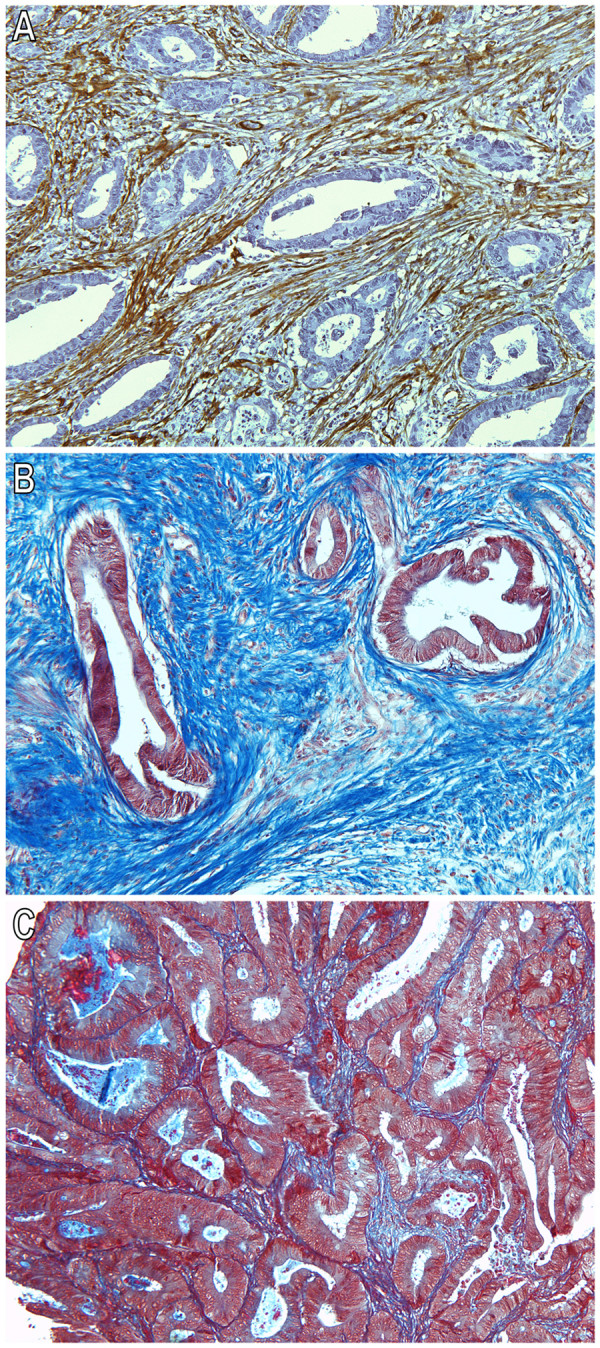
**A: Immunohistochemistry (IHC) of alpha-Smooth muscle actin (α-SMA, prediluted mouse monoclonal anti-α SMA, Abcam, Cambridge, UK) in a Stage IIIC colorectal carcinoma with high *PRL-3 *mRNA expression **[8]. Representative slide photography showing intense staining of stromal cells corresponding to CAFs. Briefly, mouse monoclonal anti-α SMA antibody was diluted 1/3 in PBS and incubated for 1 hour at room temperature. After washing steps, reaction was visualized using EnVision anti-mouse antibody system, and developed using DAB-Plus Kit (Dako, Copenhagen, Denmark). Slides were counterstained with Harry's modified hematoxylin. As a negative control, we used EnVision anti-mouse antibody system which displayed no reactivity against any antigen. Positive and negative controls were included in each assay. For IHC quantification, we evaluated non-adjacent tumoral regions to the muscular layer to avoid confusing results. Expression was evaluated according to intensity (1 = -, undetectable or + light; 2 = ++, moderate; 3 = +++, intense staining) and percentage of stain (1 = < 10%; 2 = 10–50%; 3 = > 50%). A score containing the information for both parameters [(intensity +1) × percentage] was assigned, classifying staining as "low" or "high". B: Intense deposition of collagen fibrils in a high mRNA PRL-3 expression Stage IIIC colorectal carcinoma, using Masson trichrome staining (Sigma-Aldrich). To evaluate staining we considered the percentage of tissue occupied by collagen fibrils in relation to total tissue area in each slide without taking into account the muscular layer (< 5% absence; 5–15% slight; 15–50% moderate and > 50% intense deposition). C: Slight deposition of collagen fibrils in a Stage IIIC colorectal carcinoma with previously documented low levels of mRNA *PRL-3 *expression [8].

**Figure 4:**
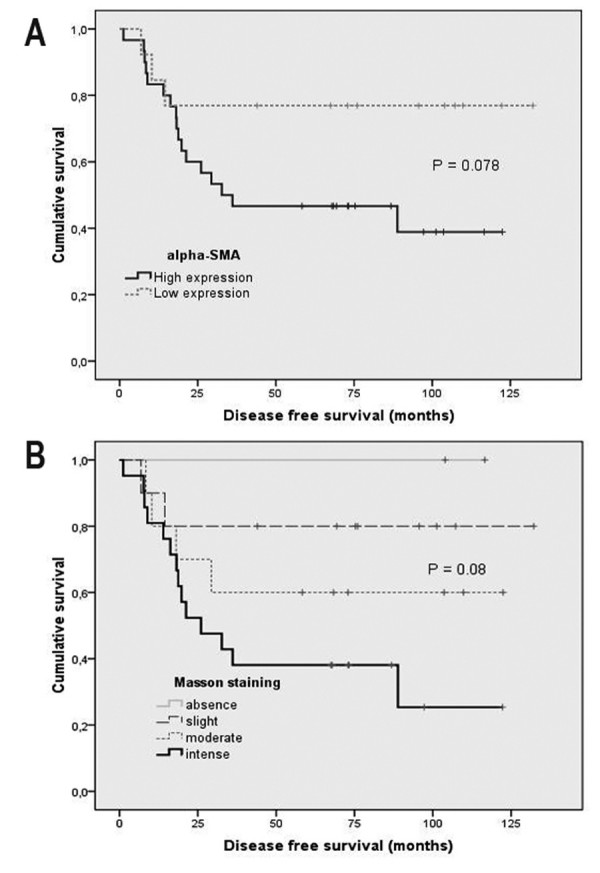
**Disease free survival Kaplan-Meier plots according to (A) alpha-smooth muscle actin (α-SMA) status and (B) Masson trichrome staining**. 43 metastasic primary colorectal tumours (22 stage IIIB and 21 stage IIIC) were obtained from the Department of Pathology of the Hospital Universitari de Bellvitge. All patients underwent radical surgical resection and received chemotherapy adjuvant treatment. P-values correspond to Log rank test and were considered significant if P ≤ 0.05. All patients gave written consent to give tumour samples to Hospital Universitari de Bellvitge tumour bank, and the ethics committee of the hospital cleared the tumour harvesting and grafting protocols.

These results, in combination with prior reports outlining PRL-3 overexpression in hepatic metastases and metastasic primary tumours [8,9] led to the hypothesis that PRL-3 expression is induced by products secreted by surrounding activated fibroblasts adjacent to the tumour compartment. We then evaluated the expression of smooth muscle actin (α-SMA), and collagen deposition (Masson staining) in a subset of 43 metastasic primary tumours (22 stage IIIB and 21 stage IIIC) for which we had paraffin-embedded tissue, from the entire 80 samples series where we previously assessed PRL-3 mRNA expression [8]. The evaluation was done by two blinded independent investigators (XS and LP). In samples with high mRNA PRL-3 expression, 85.71% (18/21) of tumours displayed high degree of infiltration of CAFs evaluated as the proportion of mesenchymal cells stained with α-SMA antibody (Chi square test, P = 0.029; Figure 3A). Moreover, the evaluation of collagen deposition revealed a high degree of fibrils in tumours with high mRNA PRL-3 expression, accounting for 71.4% with intense deposition (Figure 3B), 19.04% for moderate and 9.52% for slight or absence (Figure 3C)(Chi square test, P = 0.009). In addition, binary logistic regression showed that both high expression of α-SMA and intense and moderate Masson staining associate with tumour aggressiveness and were predictors of distant metachronous metastasis in univariate analysis (P = 0.05, OR 4.35, CI 0.99–19.12 for α-SMA; P = 0.024, OR 6.92, CI 1.29–37.05 for collagen deposition). As shown in figure 4, high expression of α-SMA and intense deposition of collagen fibrils trend with worse prognosis, although not reaching statistic significance probably due to sample size.

Hence, tumours with high mRNA PRL-3 have a more aggressive behaviour and high probability to relapse. As high primary tumour PRL-3 expression is induced by stromal factors, the abundance of myofibroblasts in cancer-associated stroma may be a useful indicator of disease recurrence after curative colorectal cancer surgery as suggested by Tsujino T *et al *[10]. Notwithstanding, for prognosis purposes is mandatory to validate the α-SMA expression and collagen deposition in larger and consecutive series in the adjuvant setting.

Crosstalk between tumour microenvironment and transformed epithelial cells plays a central role in the fate of malignant tumour cells by locally modifying signalling pathways at the primary tumour site, enhancing the migration and invasion of metastatic cells and, preparing secondary organ sites for metastatic growth [11]. More interestingly, genetic alterations in CAFs [12] and deregulation of key pathways [13] may enhance the migration and invasion through those crosstalk mechanisms. PRL-3 is key molecule involved in those processes of migration and invasion, and has been involved in the natural outgrowth of metastatic capabilities of desmoplastic tumours like colon, pancreas and breast. Here, we demonstrate that elevated PRL-3 expression, observed in colorectal primary tumours and distant secondary sites, is induced by products secreted by the surrounding activated stroma. Identification of those CAF-derived factors may inform on new stroma-targeted therapies for desmoplastic tumours.

## Abbreviations

CAF: carcinoma-associated fibroblast; CAFpt: carcinoma-associated fibroblast from primary tumour; CAFlm: carcinoma-associated fibroblast from liver metastasis; NCF: normal colonic fibroblast; FF: foreskin fibroblast; CM: conditioned media; αSMA: alpha smouth muscle actin.

## Competing interests

The authors declare that they have no competing interests.

## Authors' contributions

DGM drafted the manuscript. Moreover carried out cellular cultures, statistical analysis and coordinated the study. AA performed primary fibroblasts cultures. MB carried out PCR quantification. LP performed immunohystochemical staining of α SMA. MMI participated in tha Masson staining. XSJ provided fresh colorectal specimen and evaluated the stainings. RS carried out the management of clinical data from the colorectal database. AV provided financial support and helped to draft the manuscript. All authors read and approved the final manuscript.
